# Lunar farside-nearside asymmetry formation during magma ocean solidification

**DOI:** 10.1093/nsr/nwag424

**Published:** 2026-07-13

**Authors:** Yuantao Gu, Yue Zhang, Wenxin Ouyang, Hejiu Hui, Kai Zhao, Yongli Xue, Ziyan Han, Yuxin Chen, Zhuqi Yang, Yutao Lin, Yue Guan, Qiuli Li, Tao Yang, Gang Zeng, Huan Hu, Zongjun Yin, Suping Wu, Nan Zhang, Xiaolei Wang, Xiancai Lu, Rucheng Wang

**Affiliations:** College of Environment and Biological Engineering, Henan University of Engineering, Zhengzhou 451191, China; School of Geography and Ocean Science, Nanjing University, Nanjing 210023, China; State Key Laboratory of Critical Earth Material Cycling and Mineral Deposits & Lunar and Planetary Science Institute, School of Earth Sciences and Engineering, Nanjing University, Nanjing 210023, China; State Key Laboratory of Critical Earth Material Cycling and Mineral Deposits & Lunar and Planetary Science Institute, School of Earth Sciences and Engineering, Nanjing University, Nanjing 210023, China; State Key Laboratory of Critical Earth Material Cycling and Mineral Deposits & Lunar and Planetary Science Institute, School of Earth Sciences and Engineering, Nanjing University, Nanjing 210023, China; CAS Center for Excellence in Comparative Planetology, Hefei 230036, China; Key Laboratory of Earth and Planetary Physics, Institute of Geology and Geophysics, Chinese Academy of Sciences, Beijing 100029, China; State Key Laboratory of Critical Earth Material Cycling and Mineral Deposits & Lunar and Planetary Science Institute, School of Earth Sciences and Engineering, Nanjing University, Nanjing 210023, China; CAS Key Laboratory of Planetary Sciences, Purple Mountain Observatory, Chinese Academy of Science, Nanjing 210023, China; State Key Laboratory of Critical Earth Material Cycling and Mineral Deposits & Lunar and Planetary Science Institute, School of Earth Sciences and Engineering, Nanjing University, Nanjing 210023, China; Key Laboratory of Gemological Design and Testing, School of Jewelry and Art Design, Wuzhou University, Wuzhou 543002, China; State Key Laboratory of Critical Earth Material Cycling and Mineral Deposits & Lunar and Planetary Science Institute, School of Earth Sciences and Engineering, Nanjing University, Nanjing 210023, China; State Key Laboratory of Critical Earth Material Cycling and Mineral Deposits & Lunar and Planetary Science Institute, School of Earth Sciences and Engineering, Nanjing University, Nanjing 210023, China; State Key Laboratory of Critical Earth Material Cycling and Mineral Deposits & Lunar and Planetary Science Institute, School of Earth Sciences and Engineering, Nanjing University, Nanjing 210023, China; State Key Laboratory of Critical Earth Material Cycling and Mineral Deposits & Lunar and Planetary Science Institute, School of Earth Sciences and Engineering, Nanjing University, Nanjing 210023, China; State Key Laboratory of Critical Earth Material Cycling and Mineral Deposits & Lunar and Planetary Science Institute, School of Earth Sciences and Engineering, Nanjing University, Nanjing 210023, China; State Key Laboratory of Lithospheric and Environmental Coevolution, Institute of Geology and Geophysics, Chinese Academy of Sciences, Beijing 100029, China; State Key Laboratory of Critical Earth Material Cycling and Mineral Deposits & Lunar and Planetary Science Institute, School of Earth Sciences and Engineering, Nanjing University, Nanjing 210023, China; State Key Laboratory of Critical Earth Material Cycling and Mineral Deposits & Lunar and Planetary Science Institute, School of Earth Sciences and Engineering, Nanjing University, Nanjing 210023, China; State Key Laboratory of Critical Earth Material Cycling and Mineral Deposits & Lunar and Planetary Science Institute, School of Earth Sciences and Engineering, Nanjing University, Nanjing 210023, China; State Key Laboratory of Palaeobiology and Stratigraphy, Nanjing Institute of Geology and Palaeontology, Chinese Academy of Sciences, Nanjing 210008, China; State Key Laboratory of Palaeobiology and Stratigraphy, Nanjing Institute of Geology and Palaeontology, Chinese Academy of Sciences, Nanjing 210008, China; Key Laboratory of Orogenic Belts and Crustal Evolution, School of Earth and Space Sciences, Peking University, Beijing 100871, China; State Key Laboratory of Critical Earth Material Cycling and Mineral Deposits & Lunar and Planetary Science Institute, School of Earth Sciences and Engineering, Nanjing University, Nanjing 210023, China; State Key Laboratory of Critical Earth Material Cycling and Mineral Deposits & Lunar and Planetary Science Institute, School of Earth Sciences and Engineering, Nanjing University, Nanjing 210023, China; Frontiers Science Center for Critical Earth Material Cycling, Nanjing University, Nanjing 210023, China; State Key Laboratory of Critical Earth Material Cycling and Mineral Deposits & Lunar and Planetary Science Institute, School of Earth Sciences and Engineering, Nanjing University, Nanjing 210023, China; Frontiers Science Center for Critical Earth Material Cycling, Nanjing University, Nanjing 210023, China

**Keywords:** Chang’e-6, lunar farside-nearside asymmetry, highlands lithologies, basalts, lateral convection

## Abstract

Unraveling the origin of lunar farside-nearside asymmetry requires comparison of various lithologies from both hemispheres, a task long hindered by the absence of farside samples. Here, we present geochemical data for Chang’e 6 (CE6) lithologies from the farside, revealing systematic differences from their nearside counterparts. CE6 anorthosites are more magnesian, while CE6 Mg-suite samples contain lower potassium, rare earth element, and phosphorus (KREEP) contents. However, CE6 Mg-suite samples have Mg# values comparable to Apollo Mg-suite rocks. The mantle source of CE6 2.8-billion-year-old basalts is similar to that of Apollo low-Ti basalts. This systematic geochemical comparison of farside and nearside lithologies, which sample distinct lunar magma ocean (LMO) product layers, indicates that the mantle displays no clear compositional dichotomy. In contrast, the farside anorthositic crust is more magnesium-rich and thicker, while late-stage LMO products may be thinner on the farside. Therefore, we propose that the observed farside-nearside asymmetry originated from lateral thermal convection within a long-lived LMO on the tidally locked Moon.

## Teaser

New geochemical data from Chang’e 6 farside samples advance our understanding of the formation of the lunar nearside-farside dichotomy. Our proposed model of lateral thermal convection within the magma ocean of the tidally locked Moon provides a self-consistent mechanism accounting for the observed mantle-to-crust hemispheric variations.

## INTRODUCTION

The Moon presents striking geomorphologic and surface compositional asymmetry with the low nearside enriched in highly incompatible elements and the high farside relatively depleted in these elements [1–3]. Similarly, crustal thickness and mare volcanism distribution [3,4] also show this farside-nearside dichotomy. This hemispherical dichotomy represents a large-scale record of early planetary cooling [5]. Therefore,

the origin of this dichotomy is vital for understanding the Moon’s evolution and the solidification history of terrestrial planets [5]. Over the past decades, a variety of hypotheses have been proposed to explain its origin, including asymmetric magma ocean convection [6], asymmetry-forming impact [7], tidal dissipation [8,9], and asymmetric crustal growth [5]. These models are inconsistent, and how this lunar farside-nearside crustal asymmetry formed remains highly controversial. In addition, from a generalized perspective, the global vertical stratigraphy of lunar magma ocean (LMO) crystallization is conventionally divided into three major units: mafic cumulates, anorthositic crust, and late-stage differentiation products comprising ilmenite-bearing cumulates (IBC) and the final residual melts highly enriched in potassium, rare earth elements, and phosphorus (KREEP) [10]. It remains unknown whether this crustal asymmetry extends into the lunar mantle and, if so, the depth and nature of such a mantle dichotomy. The primary difficulty in unravelling the origin of this asymmetry is no farside samples available to be compared with the nearside samples.

A comparative study of the lunar layers between the farside and nearside is required to unravel the origin of the lunar dichotomy. Mare basalt represents the partial melting products of mafic cumulates from lunar mantle and anorthosite has been considered to be the main lithology of highlands crust [11]. Lithologies derived from mantle to crust could serve as ideal samples for comparing the lunar nearside and farside from interior to surface. In addition, plutonic Mg-suite, an important lithology of highlands crust, may have recorded the interaction between mantle and crust, and likely incorporated varying proportions of KREEP component [12,13], making it a valuable geochemical archive for multiple layers of the Moon. Nevertheless, the spatial distribution of Mg-suite rocks on the Moon remains uncertain. Whether these rocks are uniquely linked to the nearside Procellarum KREEP Terrane or have a global distribution [14–18] is still under debate. Therefore, it remains unclear whether the farside Mg-suite rocks share similar characteristics and petrogenesis with their nearside Apollo counterparts. These questions may be resolved through samples returned from the lunar farside.

**Figure 1.**
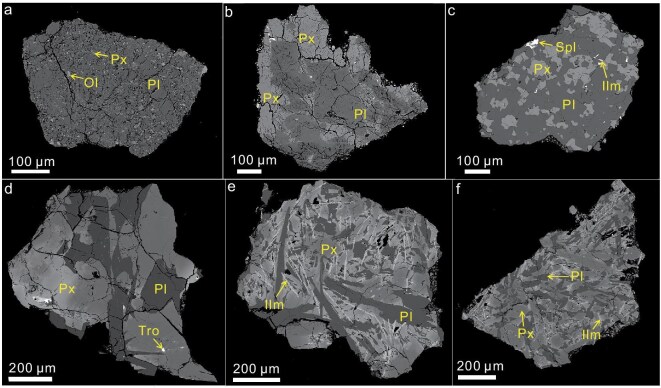
Backscattered electron images of CE6 highlands and basaltic clasts. (a) Gabbroic anorthosite (clast F30). (b) Anorthositic norite of Mg-suite (clast G05). (c) Anorthositic gabbro of Mg-suite (clast G38). (d) Basalt (clast D19). (e) Low-Ti basalt (clast B03). (f) Low-Ti basalt (clast F42). Px, pyroxene; Pl, plagioclase; Ol, olivine; Ilm, ilmenite; Tro, troilite; Spl, spinel.

The scarcity of lunar samples, especially from the farside, has severely constrained our ability to characterize the entire farside hemisphere. However, the LMO was an inherently global process [19]; therefore, samples from a single location that record distinct depths or crystallization stages of the LMO can provide a representative vertical cross-section to rigorously test whether hemispheric asymmetry was generated *ab initio* during LMO solidification. China’s Chang’e 6 (CE6) mission successfully returned lunar farside regolith samples from Apollo crater within the South Pole-Aitken (SPA) basin. Diverse lithic fragments, including breccia, anorthosite, mare basalt, and Mg-suite, have been recovered in CE6 regolith samples. These first-known farside samples provide an unprecedented opportunity to directly determine the similarities and differences between the farside and nearside from mantle to crust. Using integrated 3D texture analysis, petrographic observation, and major element analysis (Supplementary Materials), we systematically identified 37 highlands clasts (Fig. 1 and Fig. S1, Table S1) and 10 basalt clasts (Fig. 1 and Fig. S2, Table S2) from the allocated CE6 regolith sample (CE6C0200YJFM002). Although these clasts may not be representative of the entire farside, the global nature of LMO crystallization enables them to provide new constraints on the origin of lunar farside-nearside dichotomy.

## RESULTS AND DISCUSSION

We first conducted a farside-nearside geochemical comparison of highlands lithologies (anorthosite and Mg-suite) and mare basalts to delineate their similarities and differences. Subsequently, based on the distinct petrogeneses and sources of these lithologies, we performed a vertical, layer-by-layer comparison between the two hemispheres, focusing on the early mantle cumulates, anorthositic crust, and late-stage LMO products (IBC and KREEP). Finally, we propose an integrated framework to explain the origin of the lunar farside-nearside dichotomy.

### Comparison of CE6 and apollo highlands lithologies

The clasts derived from highlands crust in CE6 regolith samples are dominantly anorthosite and Mg-suite, and the latter is composed of norite and gabbro (Fig. 1, Figs S1 and S3a, Table S1). Most of the anorthosite clasts containing minor pyroxene and olivine, exhibit impact texture (Fig. 1a and Fig. S1). The identification of the farside Mg-suite clasts in CE6 samples provides compelling evidence to support the potential global occurrence of Mg-suite magmatism, challenging the notion that such magmatism was regionally restricted. Some Mg-suite clasts, such as anorthositic norite (Fig. 1b), exhibit a coarse-grained plutonic texture, similar to Apollo Mg-suite 78238 [20]. The other Mg-suite clasts present fine-grained, anhedral pyroxene as interstitial material filling the plagioclase grains (Fig. 1c and Fig. S1), similar to the fine-grained Mg-suite clasts in the impact melt rocks from the nearside [21]. These observations suggest that the CE6 highlands lithologies could have undergone impacts, which may have altered the textures of the clasts (Fig. 1c and Fig. S1). However, CE6 regolith is dominated by local lithologies and contains only trace amounts of exogenous meteoritic components [22]; thus, impact-driven material mixing is unlikely to have introduced sufficient meteoritic materials to alter primary major-element compositions. Furthermore, even if CE6 samples contain abundant impact-ejected materials, such exotic ejecta components originate almost exclusively from the lunar farside. Therefore, the major-element compositions of rock-forming minerals (plagioclase and pyroxene) in CE6 highlands lithologies record the primary chemical characteristics of the lunar farside. Accordingly, exotic components in the CE6 samples have a negligible impact on our farside-nearside major-element geochemical comparison.

Pyroxenes in CE6 anorthosite and Mg-suite are mainly low-Ca pyroxene, with some high-Ca pyroxene (Fig. S3b, Table S1). Pyroxene Mg# [molar Mg/(Mg + Fe) × 100] values (58.3–88.3) in Mg-suite are generally higher than those (55.3–74.5) in anorthosite (Fig. S3b, Table S1). Plagioclases in anorthosite and Mg-suite have high An [molar Ca/(Ca + Na + K) × 100] values, 94.1–98.6 and 90.3–98.5, respectively (Fig. S3c, Table S1). Overall, correlations between mafic mineral Mg# and plagioclase An in most CE6 highlands clasts fall within the range of Apollo highlands lithologies, with CE6 anorthosites close to high Mg# end (Fig. S3d). No alkaline-suite was observed in the allocated CE6 samples, and all Mg-suite plagioclases were clustered at high An end-member (Fig. S3d), implying depletion of alkali elements in these highlands clasts.

**Figure 2.**
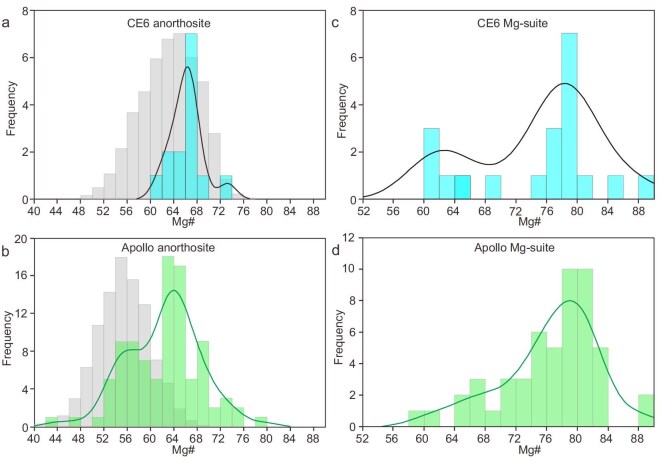
Comparison of low-Ca pyroxene Mg# distribution between CE6 and Apollo highlands lithologies. (a) Mg# distribution in CE6 anorthosites. (b) Mg# distribution in Apollo anorthosites. (c) Mg# distribution in CE6 Mg-suite rocks. (d) Mg# distribution in Apollo Mg-suite rocks. Curves represent kernel density distributions of Mg# in the respective lithologies. Gray histograms in (a) and (b) represent Mg# distributions derived from the remote-sensing data [5] from the farside and nearside, respectively. Data of CE6 lithologies are shown in Table S1 and of Apollo lithologies are from [11,55] and references therein.

Low-Ca pyroxenes in CE6 anorthosites display Mg# values ranging from 61 to 74, with a well-defined peak at ∼67 (Fig. 2a, Table S1). This peak is consistent with the peak value derived from remote-sensing data of the farside highlands (Fig. 2a). Apollo anorthosites, in contrast, exhibit a broader Mg# distribution that encompasses the range observed in CE6 anorthosites (Fig. 2a, b). Notably, the peak Mg# of CE6 anorthosites is apparently higher than the two peaks (∼56 and ∼63) observed in Apollo anorthosites (Fig. 2a, b). The low Mg# peak (∼56) in Apollo anorthosites is consistent with that derived from remote-sensing data (∼55) of the nearside highlands (Fig. 2b). The high Mg# peak (∼63) in Apollo anorthosites most likely reflects the overrepresentation of Apollo 16 anorthosites and the large number of analyses conducted on these samples. Consistently, across both remote-sensing data from the nearside highlands and measurements of Apollo anorthosites, CE6 anorthosites exhibit a higher Mg# peak value. In addition, kernel density estimation, the approximation of the density functions from the Mg# observations, suggests that Mg# is most frequently distributed at 66 in CE6 anorthosites, higher than 64 in Apollo anorthosites (Fig. 2a, b). Furthermore, a two-sample Kolmogorov-Smirnov (K-S) test of Mg# distributions shows a statistically significant difference between CE6 and Apollo anorthosites at a predetermined significance level of 0.05, with medians of 66.4 and 63.0, respectively. Nevertheless, sampling bias cannot be entirely ruled out; additional samples from the farside would help to fully explore the farside-nearside compositional difference of anorthositic crust. On the other hand, the peak Mg# represents the most frequent composition in each sample suite, which can capture the key characteristics of the respective sampling sites. Therefore, when combined with remote-sensing data, these statistical comparisons demonstrate a compositional difference between CE6 and Apollo anorthosites, implying that farside anorthosites are more magnesium-rich than their nearside counterparts.

**Figure 3.**
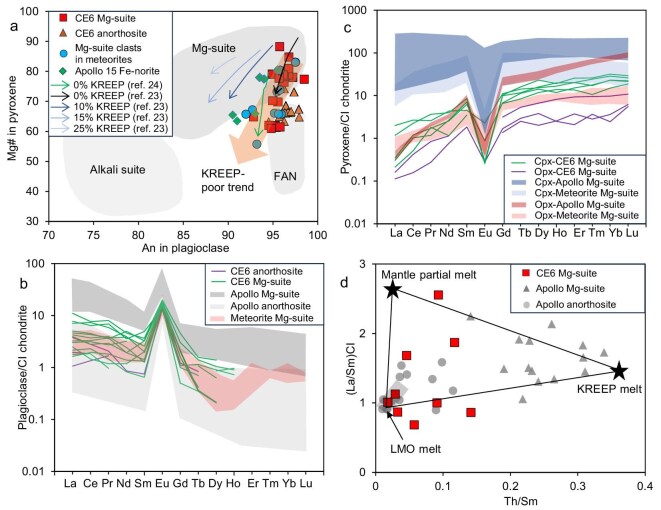
Major and trace element compositions of CE6 highlands lithologies. (a) Correlation between pyroxene Mg# and plagioclase An content in highlands clasts. Color curves represent thermodynamic modeling results for the sources of Mg-suite melts with varying amounts of KREEP [23,24]. (b) CI-chondrite normalized plagioclase REE distribution patterns in anorthosites and Mg-suite rocks. (c) CI-chondrite normalized pyroxene REE distribution patterns in Mg-suite rocks (excluding troctolite). (d) Incompatible trace element ratios in highlands lithologies. Trace element compositions of CE6 Mg-suite rocks are those of melts in equilibrium with plagioclase and pyroxene calculated using respective partition coefficients [56,57]. Chemical compositions of KREEP (potassium, REE, phosphorus) melt, mantle partial melt, and LMO (lunar magma ocean) melt are from literature [12]. Data of Mg-suite clasts in meteorites, Apollo anorthosites and Mg-suite rocks are from [11–13,15] and references therein. Cpx, clinopyroxene; Opx, orthopyroxene.

CE6 and Apollo Mg-suite rocks have similar low-Ca pyroxene Mg# range and a similar peak value of ∼79 in histograms (Fig. 2c, d). Both kernel density estimation (Fig. 2c, d) and K–S test (both Mg-suite rocks have a median of 78.0) of Mg# between CE6 and Apollo Mg-suite rocks also indicate no significant difference in Mg# between the farside and nearside Mg-suite rocks. However, CE6 Mg-suite presents a differentiation trend that deviates from the canonical trend of typical Apollo Mg-suite rocks in the correlation between pyroxene Mg# and plagioclase An content (Fig. 3a). This trend is consistent with that observed in KREEP-free Mg-suite meteorites (such as Arguin 002) and Apollo 15 Fe-noritic clasts (Fig. 3a), which may originate from KREEP-free primary magmas [23,24]. In contrast, typical Apollo Mg-suite rocks are thought to contain 10–25 wt% KREEP components [23], implying that CE6 Mg-suite likely has minimal KREEP enrichment.

Plagioclase rare earth element (REE) abundances in CE6 anorthosite fall within the range of Apollo anorthosite (Fig. 3b, Table S2). However, REE contents in both plagioclase and pyroxene of CE6 Mg-suite are lower than those of Apollo Mg-suite (Fig. 3b, c, Table S2), suggesting that CE6 Mg-suite is more depleted than Apollo Mg-suite. Furthermore, Th/Sm ratios in melts equilibrated with plagioclase and pyroxene of CE6 Mg-suite are lower than those of Apollo Mg-suite, indicating that CE6 Mg-suite is relatively KREEP-poor (Fig. 3d). Note that plagioclase and pyroxene in highlands lithologies display exceptionally low REE abundances. Impact processes can enrich pre-existing REE but are unlikely to deplete them seriously [e.g. 25,26]. The extremely REE-poor composition of CE6 Mg-suite clasts suggests that they have not been mixed with or overprinted by REE-rich impact components. Accordingly, their low-REE signature is interpreted as primary, indicating a KREEP-poor origin. On the other hand, formation of most Apollo Mg-suite rocks could have hybridized variable fractions of KREEP component [13,23]. Collectively, the KREEP-poor differentiation trend (Fig. 3a), low REE abundances in plagioclase and pyroxene (Fig. 3b, c), and low Th/Sm ratios (Fig. 3d) indicate that the CE6 Mg-suite may have experienced limited incorporation of KREEP, in contrast to the Apollo Mg-suite.

### Comparison of CE6 and nearside mare basalts

CE6 basaltic clasts typically exhibit subophitic and porphyritic textures (Fig. 1 and Fig. S2). Mineral assemblages in these clasts mainly consist of clinopyroxene, plagioclase, and ilmenite, with some accessory minerals (Fig. 1 and Fig. S2, Table S2). Major element compositions of these CE6 basaltic rock-forming minerals largely overlap with those in Apollo basalts (Fig. S4). Bulk major element compositions of these basalt clasts could be estimated using mineral modal abundances (Table S2) and chemical compositions (Table S1). Bulk TiO_2_ contents of CE6 basalts have a variation of 3.97–6.74 wt% (Table S3), falling within the range of low-Ti basalts [27]. This low-Ti range is consistent with the observation that pyroxene Ti# [molar Ti/(Ti + Cr) × 100] and Fe# [molar Fe/(Fe + Mg) × 100] in CE6 basalts present a similar correlation to that of Apollo low-Ti basalts (Fig. S4d). Pyroxene and plagioclase REE abundances of CE6 basalts are lower than those of CE5 basalts (Fig. S5a, b). Incompatible trace element ratios of the melt in equilibrium with pyroxene in CE6 basalts fall within the range of Apollo low-Ti basalts (Fig. S5c), consistent with the similar pyroxene Ti#-Fe# correlations between CE6 basalts and Apollo low-Ti basalts (Fig. S4d). The trace element modeling suggests that the parental melt of CE6 basalts (Table S4) could be generated through 2%–3% partial melting of a mantle source directly derived from the LMO [28] and subsequent fractional crystallization in the magma chamber (Fig. S5d).

The *in-situ* U-Pb isotopic compositions of different phases in five basaltic clasts (Table S3) determined by secondary ion mass spectrometer (SIMS) show that the leftmost Pb-Pb isochrons of CE6 basalt clasts F34, F29, and F42 are consistent with each other, yielding ages of 2812.8 ± 6.6 Ma, 2821 ± 15 Ma, and 2740 ± 140 Ma, respectively (Fig. S6). This consistency indicates that these clasts can be used to define an integrated Pb-Pb isochron, yielding a Pb-Pb age of 2812.1 ± 4.9 Ma (Fig. 4a), consistent with the CE6 basalt age reported in literature [29–31]. The mantle source μ value (^238^U/^204^Pb) of the CE6 basalt was determined using the lunar Pb isotope evolution model [32,33], yielding a best-fit value of 330 ± 39. This value represents one of the lowest μ values reported for all returned lunar basalts (Fig. 4c), and is consistent with previous estimates [29,30]. Plagioclase Sr isotope compositions of CE6 basalts (Table S5), analyzed by laser ablation multi-collector inductively coupled plasma mass spectrometer (LA-MC-ICP-MS), were used to calculate ^87^Rb/^86^Sr of their mantle sources. The results fall within the low-value range defined by CE5 and Apollo basalts (Fig. 4b). The low μ value (330 ± 39) and the low ^87^Rb/^86^Sr (0.002–0.019) of the CE6 basalt mantle source (Fig. 4b, c) support that this mantle source was depleted [34], consistent with the estimated REE compositions of mantle source for CE6 basalts (Fig. S5d, Table S4). It is noteworthy that the ^87^Rb/^86^Sr ratio and μ value of mantle source for CE6 basalts fall on the evolution paths of mantle sources for most Apollo low-Ti basalts (Fig. 4b, c). This isotopic similarity, together with the indistinguishable pyroxene Ti#-Fe# correlation (Fig. S4d) and trace‑element ratios (Fig. S5c) between CE6 and Apollo low‑Ti basalts, implies that these two basalt suites, originating from different lunar hemispheres, were derived from mantle sources with similar geochemical characteristics. Additionally, the mantle sources of CE6 basalts and Apollo low-Ti basalts show comparable REE distribution patterns (Fig. S5d) [35]. Collectively, multiple lines of geochemical evidence suggest that CE6 basalt may originate from a mantle source similar to that of Apollo low-Ti basalt.

**Figure:**
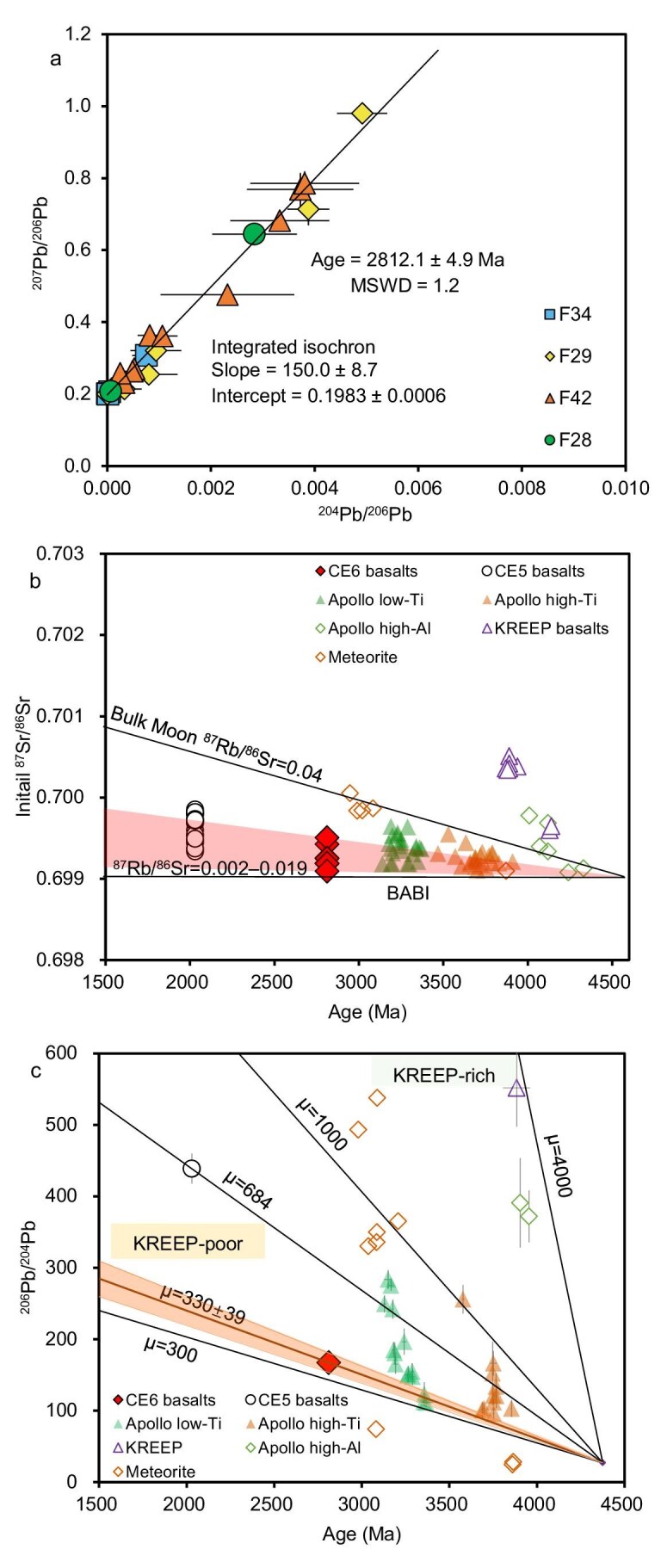
Isotope age and evolution of CE6 low-Ti basalts. (a) Pb-Pb isochron of CE6 basalts. Error bars represent 2σ uncertainties. Pb-Pb isotope composition of basaltic clasts F34, F28, F29, and F42 are used to define the leftmost Pb-Pb isochron. (b) ^87^Sr/^86^Sr isotope evolution of CE6 basalts. Initial ^87^Sr/^86^Sr ratios of CE6 basalts were calculated using an age of 2812.1 Ma (Table S5). ^87^Sr/^86^Sr of BABI (Basaltic Achondrite Best Initial) is 0.69903 [58]. (c) Pb-Pb isotope evolution of CE6 basalts. Dashed lines denote Pb isotope evolution of source using a two-stage model [32]. Data of Apollo basalts, CE5 basalts, and lunar meteorites are from [33,47] and references therein.

Lunar monthly tidal response and basalt crystallization temperatures indicate that the farside mantle is cooler than the nearside mantle, with a temperature difference of ∼100–200 K [36,37]. The enrichment of KREEP on the nearside supports the hypothesis of a hotter nearside mantle and could sustain a warmer thermal regime [36]. In contrast, such a heat source was diminished on the cooler farside, and the parental magma of CE6 basalts exhibits no KREEP signature (Fig. 4b, c). Therefore, the farside mare volcanism recorded by the ∼2.8 Ga CE6 basalts may involve distinct thermal mechanisms from the nearside volcanism represented by Apollo and CE5 basalts. Magmatic underplating beneath the shallow IBC, where partial melts from the deep mantle ascended via magmatic dikes, could have provided the thermal impetus for CE6 basaltic volcanism [38,39]. The active lunar dynamo recorded by CE-6 basalts provides key evidence for persistent deep mantle convection on the farside [40], indicating that sufficient internal heat was sustained in the lunar farside interior to support such magmatic activity.

CE6 lithic clasts were collected from the lunar regolith, where crustal and mantle-derived lithologies have experienced prolonged space weathering and impact processes [22] and may exhibit local lithological heterogeneity. However, the basaltic clasts analyzed in this study exhibit excellent preservation of primary igneous textures (Fig. 1 and Fig. S2) and show no petrographic evidence of impact melting or shock metamorphism sufficient to alter their isotopic compositions. For anorthosite and Mg-suite clasts, as demonstrated above, surface processes including impact excavation and mixing have produced only negligible modifications to their major-element compositions. Moreover, modifying agents and processes are almost exclusively from the lunar farside, which further minimizes potential bias in the global nearside-farside geochemical comparison. In addition, the low abundances of trace elements in the Mg-suite clasts (Fig. 3b, c) show no evidence of significant secondary overprinting, confirming that these rocks retain their primary magmatic signatures. Collectively, these observations demonstrate that the CE6 clasts analyzed in this study are suitable for nearside-farside geochemical comparisons, providing a solid foundation for the following discussion.

### Hemispheric contrasts from the mantle to the crust

It has been demonstrated that lunar nearside mantle is heterogeneous, as evidenced by different mineral assemblages and trace element compositions among various Apollo basalts [35]. On the other hand, similar depleted mantle sources (Fig. 4b, c) of CE6 basalts and Apollo low-Ti basalts suggest that the early mantle cumulates may not exhibit substantial differences between the farside and nearside, with clear geochemical overlap between the two hemispheres. In addition, multiple petrogenetic lines of evidence demonstrate that Mg-suite formation is fundamentally linked to lunar crust-mantle interaction initiated by partial melting of early mantle cumulates, although the detailed mechanism remains debated [13,15,24]. The primitive anorthositic crust consisted almost exclusively of plagioclase and contained negligible amounts of mafic minerals [41]. Therefore, the abundant mafic minerals in Mg-suite rocks must have been derived from the underlying mantle cumulates rather than the overlying anorthositic crust. Accordingly, the Mg# of Mg-suite rocks may reflect that of early mantle cumulates. Similar Mg# values between CE6 and Apollo Mg-suite rocks (Fig. 2c, d) therefore demonstrate that the early mantle cumulates on the farside and nearside may have similar Mg#. Collectively, these lines of evidence indicate that the early mantle cumulates crystallized from LMO may not have developed a farside-nearside compositional asymmetry that observed in the lunar crust. Lunar mantle heterogeneity may not exhibit a distinct farside-nearside lateral dichotomy. We propose this as a provisional hypothesis, with future geochemical data expected to assess any compositional asymmetry between the nearside and farside mantle. In contrast, lunar mantle exhibited thermal asymmetry between the farside and nearside during the period of 2–4 Ga [36,37]. This thermal asymmetry may arise from the heterogeneous distribution of KREEP-rich materials, as supported by remote sensing observations [1]. Alternatively, it may originate from the initial thermal difference between the farside and nearside [36], potentially driven by companion-moon accretion [7] or enhanced tidal heating that slowed nearside cooling [9]. Tidal locking of the Moon established permanent nearside and farside hemispheres of the Moon. When this locking occurred early [42], the Earth-Moon distance was much smaller than today, so tidal heating concentrated on the nearside would have generated a strong hemispheric thermal gradient [9,36,37]. This primordial thermal asymmetry was thereby imprinted and may have persisted to influence LMO evolution, such as asymmetric crustal growth [5] and asymmetric magma ocean convection [6]. In summary, lunar mantle may not display a distinct farside-nearside compositional dichotomy, yet it exhibits pronounced thermal asymmetry that likely originated from tidal heating.

Plagioclase saturation and subsequent flotation in the dense LMO initiated the formation of the primary anorthositic crust [43]. Mafic minerals that coexist with plagioclase in these anorthosites become progressively Fe-rich during LMO evolution [5] and thus their Mg# may record the crystallization sequence of these mafic minerals throughout the LMO solidification. Experimental simulations indicate that low-Ca pyroxene crystallized at the early stage of crust formation yields Mg# values of ∼80–84, corresponding to the pyroxene compositions preserved in the most primitive anorthosite crust [43]. Furthermore, the Mg# range observed on the farside corresponds to a residual melt fraction (*F*) of 0.18–0.07, whereas the nearside yields lower *F* values of 0.14–0.06 [43], reflecting crystallization from more evolved LMO melts. This contrast supports that the nearside crust formation via plagioclase flotation occurred later than that on the farside, with partial temporal overlap. The experimental results are consistent with the farside-nearside comparative analyses of anorthosites (Fig. 2a, b). Specifically, the higher Mg# peak of low-Ca pyroxenes in CE6 anorthosites (Fig. 2a, b) implies that the farside crust generally formed from a more primitive magma ocean residue than the nearside crust. This ground-truth difference, combined with supporting remote-sensing data and experimental simulations, demonstrates a clear compositional difference of anorthositic crust between the farside and nearside. The canonical LMO model posits that anorthositic crust formed from buoyant plagioclase crystallized from a global magma ocean [28,43,44]. Accordingly, the farside-nearside difference in anorthosites could be attributed to solidification of the LMO. The serial magmatism model could also generate magnesian anorthosites (Mg# > 70) [45]; however, it cannot account for the observed crustal thickness difference [3] between the farside and nearside, a feature interpreted to reflect the LMO solidification process [5,8]. These findings collectively suggest that the farside-nearside asymmetry of anorthositic crust likely originated from LMO solidification and has been preserved throughout the subsequent geologic history of the Moon.

The REE enrichment observed in Apollo Mg-suite is generally attributed to the involvement of KREEP component [12,13], a late-stage residual melt of LMO thought to have formed a global layer between the anorthositic crust and underlying ultramafic mantle cumulates [12,13]. Accordingly, the parental magma of Mg-suite has been produced from a hybrid source composed of mafic cumulates, KREEP, and anorthositic crust [12,13]. However, recent studies have shown that Mg-suite magmatism may not require a KREEP component and can form via KREEP-free petrogenetic pathways [14,15,18,46]. Low‑degree partial melting of deep, primitive, and KREEP-poor cumulates, followed by interaction with anorthositic crust during magma ascent (e.g. magma-wallrock interaction, assimilation, or hybridization) [15,18,24], may represent an important mechanism to generate KREEP-poor Mg-suite rocks. Therefore, KREEP component is not a prerequisite for all Mg-suite magmatism. Similarly, the KREEP-poor signature recorded in CE6 Mg-suite rocks (Fig. 3) reflects limited involvement of KREEP in their parental magma. This provides direct petrological support for a KREEP-independent origin of Mg-suite magmatism. Alternatively, CE6 Mg-suite rocks may have formed through a mechanism similar to that for Apollo Mg-suite samples, i.e. the parental magma incorporated variable proportions of KREEP components at the base of crust [12,13]. The KREEP-poor signature in CE6 Mg-suite rocks may reveal a difference in the KREEP distribution between the farside and nearside. It is therefore plausible that the KREEP-rich layer on the farside was thinner or less accessible than that on the nearside. Overall, the pronounced KREEP-depletion in CE6 Mg-suite rocks suggests that either the magmatism was KREEP-independent, or the distribution of KREEP-rich layer may be asymmetric between the farside and nearside.

The production of Mg-suite melts may be driven by mantle overturn, accompanied by the downwelling of IBC and the ascent of hot mafic cumulates [12,14]. However, degree-1 downwelling of IBC cannot induce global Mg-suite magmatism [14] as supported by CE6 and Apollo Mg-suite lithology. This implies that IBC may be heterogeneously distributed within the lunar interior. In addition, remote sensing observations indicate that TiO_2_ contents on the farside surface are generally lower than those on the nearside, which can be attributed to the asymmetric distribution of IBC derived from the LMO [1,2,39]. A comparison of the mantle sources of CE5 and CE6 basalts has demonstrated that the uppermost mantle of the nearside may have hosted a thicker and more ilmenite-rich IBC layer than that of the farside, possibly resulting from asymmetrical LMO solidification [39]. This hemispheric asymmetry of IBC distribution has possibly caused that the high-Ti mantle sources on the farside may be rarer than that on the nearside, consistent with that CE6 basalts were not derived from a high-Ti mantle source (Fig. S5c, d). On the other hand, the LMO model predicts that the IBC layer formed following the formation of anorthositic crust but preceding both KREEP layer formation and complete solidification of the LMO [14]. This temporal offset allowed the dense IBC layer to become gravitationally unstable and initiate mantle overturn before the LMO was fully solidified. Consequently, IBC and KREEP may have become geochemically decoupled such that the nearside Mg-suite magmatism likely assimilated or mixed KREEP. Nevertheless, the non-KREEP mantle sources of CE5 basalts [47] and Apollo 15 Fe-norite [15] suggest that the KREEP layer may be heterogeneously distributed within the lunar interior.

In summary, each layer of the Moon, from the mantle to the crust, exhibits distinct characteristics between the farside and nearside. Specifically, the mantle is characterized by thermal asymmetry rather than compositional dichotomy; the anorthositic crust displays compositional asymmetry; and late-stage LMO products, namely IBC and KREEP, are asymmetrically enriched on the nearside. Given that all these lunar layers (from the interior to the surface) formed during different crystallization stages of the LMO, the observed hemispheric discrepancies are therefore likely to have originated during the solidification of the LMO.

### Origin of lunar farside-nearside asymmetry

**Figure 5.**
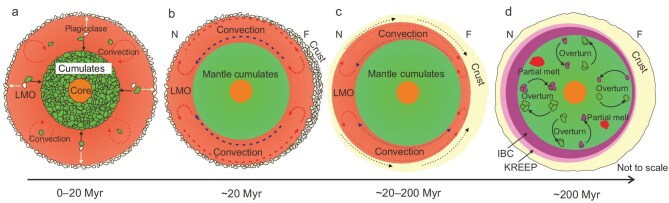
Formation of lunar farside-nearside asymmetry. (a) LMO cooled rapidly, resulting in formation of early mantle cumulates before the rotation of the Moon was synchronized. Plagioclase floated to the LMO surface to form anorthositic crust, which could have slowed the LMO solidification. (b) Thermal difference between the farside and nearside after establishment of synchronous rotation [42] may have initialized lateral convection in the magma ocean. (c) Lateral thermal convection likely moved anorthositic crust toward farside, which resulted in thickening of anorthositic crust on the farside and migration of the LMO melt residue to the nearside. (d) Cooling of the LMO residue may have formed thin or inaccessible IBC and KREEP layers on the farside and thick layers on the nearside, with both layers being internally heterogeneous. N, nearside; F, farside.

Solidification of the LMO driven solely by secular cooling could result in global differentiation of the lunar interior, compositional differences at different depths, but no hemispheric difference at each layer of the Moon (Fig. 5a). However, synchronous rotation of the Moon established at ∼20 Myr after the Moon’s formation [42] could have created lateral convection in the LMO, which may have lasted for a prolonged period of ∼200 Myr [48,49]. We propose a scenario in which lateral thermal convection initiated by tidal heating during LMO solidification may have generated hemispheric asymmetries for the late LMO products (Fig. 5). Accordingly, the early crystallization of the LMO would not have generated hemispheric differences in mantle cumulates prior to the onset of synchronous rotation (Fig. 5a). This inference is supported by our observation that the early mantle cumulates show no compositional asymmetry, as reflected by the similar Mg# values of Mg-suite rocks between the farside and nearside (Fig. 2c, d) and the comparable mantle sources of basalts from both hemispheres (Fig. 4). Furthermore, a globally homogeneous anorthositic crust may have formed rapidly during the early LMO crystallization (Fig. 5a) [44]. This early crust, which has low thermal conductivity, could have prolonged the solidification duration of the LMO [44], consistent with a long-lived LMO [48,49].

Tidal heating from Earth on the tidally synchronized Moon not only prolonged the LMO solidification [44], but also led to a hotter nearside relative to the farside [8,9]. This is consistent with the farside-nearside mantle thermal asymmetry [36,37]. Such farside-nearside thermal difference may have driven lateral convection in the LMO beneath the anorthositic crust (Fig. 5b). In addition, the lunar anorthositic crust may be in a soft-shell regime during LMO solidification [9]. Consequently, the lateral convection beneath the crust may have continuously moved floating anorthositic crust from the nearside toward the farside (Fig. 5b). The transport of early-formed anorthositic crust toward the farside produced a more magnesium-rich farside crust, because early anorthosite crystallized from a more primitive (less evolved) LMO than later-formed anorthosite [5]. The higher Mg# of farside anorthosites revealed by sample comparison (Fig. 2a, b), remote-sensing data [5], and experimental simulations [43] is fully consistent with this model for lateral migration of early floating anorthosite, although it does not provide a unique explanation for this global compositional asymmetry. The accumulation of anorthosite on the farside further led to hemispherically asymmetric crustal thickness (Fig. 5b, c), consistent with the observation that the farside anorthositic crust is much thicker than that on the nearside [3]. Accordingly, the thickened farside crust could result in a thinner subsurface magma ocean on the farside compared with the nearside (Fig. 5c) [8]. This process was likely maintained by the lateral convection, which transported residual LMO melt toward the nearside (Fig. 5c). Therefore, both the IBC and KREEP layers on the farside could be generally thinner or less accessible relative to the nearside (Fig. 5d), with each layer exhibiting internal heterogeneity. The surface abundances of Ti and Th on the Moon support this asymmetry in late-stage LMO products [1,2]. In this context, the KREEP-poor signatures of CE6 Mg-suite samples (Fig. 3) and basalts (Fig. 4) are consistent with a thin or geochemically inaccessible KREEP layer on the farside. It should be noted, however, that these signatures may alternatively reflect either a KREEP‑independent magmatic origin for the Mg-suite clasts or the spatial heterogeneity in the global KREEP layer, which exhibits lateral variations between the two hemispheres. The currently limited CE6 dataset does not permit definitive discrimination between these two competing hypotheses. Accordingly, we emphasize that the proposed asymmetric distribution of late-stage LMO differentiation products within our lateral convection model requires verification with additional farside samples. Nevertheless, the integrated geodynamic framework proposed here provides a plausible and self-consistent explanation for the observed hemispheric asymmetries spanning from the mantle to the crust.

The SPA basin presents an elliptical geomorphic outline with its major axis nearly parallel to the north-south direction [50]. Such an elliptical shape could have originated from an oblique impact [50]. Alternatively, the SPA basin may have initially been circular but was subsequently deformed into an elliptical structure by lateral crust movement driven by underlying convection. This mechanism is consistent with the observation that major axis of the SPA basin is roughly perpendicular to the inferred nearside-farside transport direction of anorthositic crust (Fig. 5c). This interpretation implies that the SPA basin formed during LMO solidification [51]. Under a steep thermal gradient (50 K/km) [52], crustal materials with weak rheology may not have been sufficiently rigid to resist deformation associated with large-scale lateral crustal movement.

In summary, CE6 regolith samples reveal that the farside anorthosites have higher Mg# values than their nearside counterparts (Fig. 2a, b). Compared with Apollo Mg-suite samples, CE6 Mg-suite clasts exhibit similar Mg# values (Fig. 2c, d) but lack KREEP components (Fig. 3). CE6 mare basalts share a comparable mantle source with Apollo low-Ti basalts (Fig. 4). Combined with previous remote-sensing observations and experimental simulations [1,5,43], the farside-nearside comparisons of these lithologies, which formed from different LMO product layers, indicate that the lunar mantle, crust, and late-stage LMO products (IBC and KREEP) each exhibit different characteristics for the hemispheric asymmetry. The mantle presents no clear compositional dichotomy but thermal asymmetry with a cooler farside mantle [36,37]. In contrast, the anorthositic crust displays clear compositional asymmetry, marked by more magnesium-rich anorthositic crust on the farside. Late-stage LMO products likely show asymmetric distributions, with thinner IBC and KREEP layers on the farside. We present a self-consistent model that reconciles the observed hemispheric variations from the mantle to the crust. This model is anchored by lateral thermal convection operating within the magma ocean of the tidally locked early Moon (Fig. 5). Such lateral convection process may operate on any planetary body whose rotation is synchronized with its host. The surface and crustal structure of such a planetary body may record this lateral convection process. Notably, the ice shell thickness patterns observed on the synchronous icy moons, such as Europa [53] and Titan [54], may be explained by lateral convection process.

## Supplementary Material

nwag424_Supplemental_Files

## References

[1] Lawrence D, Feldman W, Barraclough B et al. Global elemental maps of the Moon: the Lunar Prospector gamma-ray spectrometer. Science 1998; 281: 1484–9. 10.1126/science.281.5382.1484 9727970

[2] Lucey PG, Spudis PD, Zuber M et al. Topographic-compositional units on the Moon and the early evolution of the lunar crust. Science 1994; 266: 1855–8. 10.1126/science.266.5192.1855 17737081

[3] Wieczorek MA, Neumann GA, Nimmo F et al. The crust of the Moon as seen by GRAIL. Science 2013; 339: 671–5. 10.1126/science.1231530 23223394 PMC6693503

[4] Zuber MT, Smith DE, Lemoine FG et al. The shape and internal structure of the Moon from the Clementine mission. Science 1994; 266: 1839–43. 10.1126/science.266.5192.1839 17737077

[5] Ohtake M, Takeda H, Matsunaga T et al. Asymmetric crustal growth on the Moon indicated by primitive farside highland materials. Nat Geosci 2012; 5: 384–8. 10.1038/ngeo1458

[6] Loper D, Werner C. On lunar asymmetries 1. Tilted convection and crustal asymmetry. J Geophys Res 2002; 107: 5046. 10.1029/2000JE001441

[7] Jutzi M, Asphaug E. Forming the lunar farside highlands by accretion of a companion moon. Nature 2011; 476: 69–72. 10.1038/nature10289 21814278

[8] Garrick-Bethell I, Nimmo F, Wieczorek MA. Structure and formation of the lunar farside highlands. Science 2010; 330: 949–51. 10.1126/science.1193424 21071665

[9] Quillen AC, Martini L, Nakajima M. Near/far side asymmetry in the tidally heated Moon. Icarus 2019; 329: 182–96. 10.1016/j.icarus.2019.04.010 32934397 PMC7489467

[10] Moriarty III DP, Dygert N, Valencia SN et al. The search for lunar mantle rocks exposed on the surface of the Moon. Nat Commun 2021; 12: 4659. 10.1038/s41467-021-24626-3 34344883 PMC8333336

[11] Papike JJ, Ryder G, Shearer CK. Lunar samples. Rev Mineral Geochem 1998; 36: 5–1–5–234.

[12] Pernet-Fisher JF, Deloule E, Joy KH. Evidence of chemical heterogeneity within lunar anorthosite parental magmas. Geochim Cosmochim Acta 2019; 266: 109–30. 10.1016/j.gca.2019.03.033

[13] Shearer CK, Elardo SM, Petro NE et al. Origin of the lunar highlands Mg-suite: an integrated petrology, geochemistry, chronology, and remote sensing perspective. Am Mineral 2015; 100: 294–325. 10.2138/am-2015-4817

[14] Prissel TC, Zhang N, Jackson CRM et al. Rapid transition from primary to secondary crust building on the Moon explained by mantle overturn. Nat Commun 2023; 14: 5002. 10.1038/s41467-023-40751-7 37591857 PMC10435462

[15] Wang Z, Tian W, Wang W-RZ et al. Genesis and timing of KREEP-free lunar Mg-suite magmatism indicated by the first norite meteorite Arguin 002. Commun Earth Environ 2025; 6: 170. 10.1038/s43247-025-02086-7

[16] Pieters CM, Hanna KD, Cheek L et al. The distribution of Mg-spinel across the Moon and constraints on crustal origin. Am Mineral 2014; 99: 1893–910. 10.2138/am-2014-4776

[17] Prissel TC, Parman SW, Jackson CRM et al. Pink Moon: the petrogenesis of pink spinel anorthosites and implications concerning Mg-suite magmatism. Earth Planet Sci Lett 2014; 403: 144–56. 10.1016/j.epsl.2014.06.027

[18] Sheikh D, Ruzicka AM, Hutson ML. “Ground truth” occurrence of Pink Spinel Anorthosite (PSA) as clasts in lunar meteorite Northwest Africa (NWA) 15500: chemical evidence for a genetic relationship with lunar highlands Mg-suite and formation by magma–wallrock interactions. Meteorit Planetary Sci 2025; 60: 206–24. 10.1111/maps.14298

[19] Warren PH . The magma ocean concept and lunar evolution. Annu Rev Earth Planet Sci 1985; 13: 201–40. 10.1146/annurev.ea.13.050185.001221

[20] Zhang B, Lin Y, Moser DE et al. Timing of lunar Mg-suite magmatism constrained by SIMS U-Pb dating of Apollo norite 78238. Earth Planet Sci Lett 2021; 569: 117046. 10.1016/j.epsl.2021.117046

[21] Shearer CK, Moriarty DP, Simon SB et al. Where Is the lunar mantle and deep crust at Crisium? A perspective from the Luna 20 samples. JGR Planets 2023; 128: 147–206. 10.1029/2022JE007409

[22] Wang Z, Li Y, Li J et al. Chemical compositions of Chang’e-6 lunar soil and substantial addition of noritic crust ejecta from Apollo basin. Geology 2025; 53: 557–61. 10.1130/G53086.1

[23] Elardo SM, Astudillo Manosalva DF. Complexity and ambiguity in the relationships between major lunar crustal lithologies and meteoritic clasts inferred from major and trace element modeling. Geochim Cosmochim Acta 2023; 354: 13–26. 10.1016/j.gca.2023.05.020

[24] Prissel TC, Gross J. On the petrogenesis of lunar troctolites: new insights into cumulate mantle overturn & mantle exposures in impact basins. Earth Planet Sci Lett 2020; 551: 116531. 10.1016/j.epsl.2020.116531

[25] Gnos E, Hofmann BA, Al-Kathiri A et al. Pinpointing the source of a lunar meteorite: implications for the evolution of the Moon. Science 2004; 305: 657–9. 10.1126/science.1099397 15286369

[26] Pernet-Fisher JF, Joy KH, Martin DJP et al. Assessing the shock state of the lunar highlands: implications for the petrogenesis and chronology of crustal anorthosites. Sci Rep 2017; 7: 5888. 10.1038/s41598-017-06134-x 28724931 PMC5517601

[27] Taylor GJ, WatTen PH, Ryder G et al. Lunar Rocks. In: Heiken GH, Vaniman DT, French BV (ed) Lunar Sourcebook: A User’s Guide to the Moon. Cambridge: Cambridge University Press, 1991, 183–284.

[28] Snyder GA, Taylor LA, Neal CR. A chemical model for generating the sources of mare basalts: combined equilibrium and fractional crystallization of the lunar magmasphere. Geochim Cosmochim Acta 1992; 56: 3809–23. 10.1016/0016-7037(92)90172-F

[29] Cui Z, Yang Q, Zhang Y-Q et al. A sample of the Moon’s far side retrieved by Chang’e-6 contains 2.83-billion-year-old basalt. Science 2024; 386: 1395–9. 10.1126/science.adt1093 39545330

[30] Zhang QWL, Yang MH, Li QL et al. Lunar farside volcanism 2.8 billion years ago from Chang’e-6 basalts. Nature 2025; 643: 356–60. 10.1038/s41586-024-08382-0 39547260 PMC12240805

[31] Che X, Long T, Nemchin A et al. Isotopic and compositional constraints on the source of basalt collected from the lunar farside. Science 2025; 387: 1306–10. 10.1126/science.adt3332 40014691

[32] Snape JF, Nemchin AA, Bellucci JJ et al. Lunar basalt chronology, mantle differentiation and implications for determining the age of the Moon. Earth Planet Sci Lett 2016; 451: 149–58. 10.1016/j.epsl.2016.07.026

[33] Li QL, Zhou Q, Liu Y et al. Two-billion-year-old volcanism on the Moon from Chang’e-5 basalts. Nature 2021; 600: 54–8. 10.1038/s41586-021-04100-2 34666338 PMC8636262

[34] Zhou Q, Yang W, Chu Z et al. Ultra-depleted mantle source of basalts from the South Pole-Aitken basin. Nature 2025; 643: 371–5. 10.1038/s41586-025-09131-7 40634740 PMC12240832

[35] Hallis L, Anand M, Strekopytov S. Trace-element modelling of mare basalt parental melts: implications for a heterogeneous lunar mantle. Geochim Cosmochim Acta 2014; 134: 289–316. 10.1016/j.gca.2014.01.012

[36] He S, Li Y, Zhu X et al. A relatively cool lunar farside mantle inferred from Chang’e-6 basalts and remote sensing. Nat Geosci 2025; 18: 1103–8. 10.1038/s41561-025-01815-z

[37] Park RS, Berne A, Konopliv AS et al. Thermal asymmetry in the Moon’s mantle inferred from monthly tidal response. Nature 2025; 641: 1188–92. 10.1038/s41586-025-08949-5 40369068 PMC12119328

[38] Head JW, Wilson L, Hiesinger H et al. Lunar mmare basaltic volcanism: volcanic features and emplacement processes. Rev Mineral Geochem 2023; 89: 453–507. 10.2138/rmg.2023.89.11

[39] Wang C, Qian Y, Wang J et al. The source and thermal driver of young (<3.0 Ga) lunar volcanism. Sci Adv 2025; 11: eadv9085. 10.1126/sciadv.adv9085 40845096 PMC12372884

[40] Cai S, Qi K, Yang S et al. A reinforced lunar dynamo recorded by Chang’e-6 farside basalt. Nature 2025; 643: 361–5. 10.1038/s41586-024-08526-2 39701132 PMC12240856

[41] Lucey P, Korotev RL, Gillis JJ et al. Understanding the lunar surface and space-Moon interactions. Rev Mineral Geochem 2006; 60: 83–219. 10.2138/rmg.2006.60.2

[42] Murray CD, Dermott SF. Solar System Dynamics. Cambridge: Cambridge University Press, 1999.

[43] Charlier B, Grove TL, Namur O et al. Crystallization of the lunar magma ocean and the primordial mantle-crust differentiation of the Moon. Geochim Cosmochim Acta 2018; 234: 50–69. 10.1016/j.gca.2018.05.006

[44] Elkins-Tanton LT, Burgess S, Yin Q-Z. The lunar magma ocean: reconciling the solidification process with lunar petrology and geochronology. Earth Planet Sci Lett 2011; 304: 326–36. 10.1016/j.epsl.2011.02.004

[45] Gross J, Treiman AH, Mercer CN. Lunar feldspathic meteorites: constraints on the geology of the lunar highlands, and the origin of the lunar crust. Earth Planet Sci Lett 2014; 388: 318–28. 10.1016/j.epsl.2013.12.006

[46] Yen C, Deligny C, Jolliff B et al. Magnesian-suite volcanism and ancient crust building on the Moon 4.25 billion years ago. Earth Planet Sci Lett 2025; 662: 119395. 10.1016/j.epsl.2025.119395

[47] Tian HC, Wang H, Chen Y et al. Non-KREEP origin for Chang’e-5 basalts in the Procellarum KREEP Terrane. Nature 2021; 600: 59–63. 10.1038/s41586-021-04119-5 34666339 PMC8636255

[48] Borg LE, Connelly JN, Boyet M et al. Chronological evidence that the Moon is either young or did not have a global magma ocean. Nature 2011; 477: 70–2. 10.1038/nature10328 21849974

[49] Maurice M, Tosi N, Schwinger S et al. A long-lived magma ocean on a young Moon. Sci Adv 2020; 6: eaba8949. 10.1126/sciadv.aba8949 32695879 PMC7351470

[50] Garrick-Bethell I, Zuber MT. Elliptical structure of the lunar South Pole-Aitken basin. Icarus 2009; 204: 399–408. 10.1016/j.icarus.2009.05.032

[51] Evans AJ, Andrews-Hanna JC, Head JW et al. Reexamination of early lunar chronology with GRAIL data: terranes, basins, and impact fluxes. JGR Planets 2018; 123: 1596–617. 10.1029/2017JE005421

[52] Trowbridge AJ, Johnson BC, Freed AM et al. Why the lunar South Pole-Aitken Basin is not a mascon. Icarus 2020; 352: 113995. 10.1016/j.icarus.2020.113995

[53] Nimmo F, Thomas PC, Pappalardo RT et al. The global shape of Europa: constraints on lateral shell thickness variations. Icarus 2007; 191: 183–92. 10.1016/j.icarus.2007.04.021

[54] Zebker HA, Stiles B, Hensley S et al. Size and shape of Saturn’s moon Titan. Science 2009; 324: 921–3. 10.1126/science.1168905 19342551

[55] Torcivia M, Neal C. Unraveling the components within Apollo 16 ferroan anorthosite suite cataclastic anorthosite sample 60025: implications for the lunar magma ocean model. JGR Planets 2022; 127: e2020JE006799. 10.1029/2020JE006799

[56] Hauri EH, Wagner TP, Grove TL. Experimental and natural partitioning of Th, U, Pb and other trace elements between garnet, clinopyroxene and basaltic melts. Chem Geol 1994; 117: 149–66. 10.1016/0009-2541(94)90126-0

[57] Papike JJ, Fowler GW, Shearer CK et al. Ion microprobe investigation of plagioclase and orthopyroxene from lunar Mg-suite norites: implications for calculating parental melt REE concentrations and for assessing postcrystallization REE redistribution. Geochim Cosmochim Acta 1996; 60: 3967–78. 10.1016/0016-7037(96)00212-8

[58] Nyquist L . Lunar Rb-Sr chronology. Phys Chem Earth 1977; 10: 103–42.

