# Supplementary material for: Lunar farside-nearside asymmetry formation during magma ocean solidification
Source: Natl Sci Rev. 2026 Jul 13;13(15):nwag424. doi: 10.1093/nsr/nwag424 (PMC13455602; doi:10.1093/nsr/nwag424)
Supplement: Supplementary file 1 [file nwag424_supplemental_files.zip › 20260706 Supplementary Materials.docx]

**Supplementary Materials for**

**Lunar** **farside-nearside** **asymmetry formation during magma ocean solidification**

**This file includes:**

**Materials and Methods**

Sample preparation

3D texture analysis

Petrographic observation

Major element analysis

Trace element analysis

U-Pb isotope analysis

In-situ Sr isotope analysis

Trace-element modelling of petrogenesis

**Figs. S1 to S6**

Fig. S1 Backscattered electron images of CE6 highlands lithologies.

Fig. S2 Backscattered electron images of CE6 low-Ti basalts.

Fig. S3 Mineral modal abundances and chemical compositions of CE6 highlands lithologies.

Fig. S4 Mineral modal abundances and chemical compositions of CE6 low-Ti basalts.

Fig. S5 CI-chondrite normalized REE distribution patterns of CE6 low-Ti basalts.

Fig. S6 Pb-Pb isochron ages of CE6 low-Ti basalts.

**Tables S1 to S5**

Table S1 Mineral modes, plagioclase An, and mafic mineral Mg# in CE6 highlands lithologies.

Table S2 Mineral modes of CE6 low-Ti basalts.

Table S3 Bulk clast compositions of CE6 low-Ti basalts.

Table S4 REE compositions of parental melt and mantle source for CE6 low-Ti basalts.

Table S5 Sr isotope compositions of plagioclase in CE6 low-Ti basalts.

**Other** **Supplementary Materials for this manuscript include the following:**

Supplementary Videos 1 to 3

Supplementary Tables S1 to S4

**Materials and Methods**

**Sample preparation**

The lunar regolith sample (CE6C0200YJFM002) used in this study was scooped by the robotic arm on board the CE6 lander. A total of 193 fragments were handpicked under a binocular microscope. Fifty of these fragments were examined using X-ray tomographic microscopy (XRM) to assess 3D mineral distribution. All the fragments were embedded in epoxy resin and polished to seven thick sections for further analyses using field-emission scanning electron microscope (FE-SEM), electron probe microanalyzer (EPMA), laser ablation inductively coupled plasma mass spectrometer (LA-ICP-MS), secondary ion mass spectrometer (SIMS), and laser ablation multi-collector inductively coupled plasma mass spectrometer (LA-MC-ICP-MS).

**3D texture analysis**

Fifty large lithic fragments from CE6 samples were selected for XRM analyses. Volume digital images for each entire fragment were obtained using a Zeiss Xradia 620 Versa XRM at Nanjing Institute of Geology and Paleontology, Chinese Academy of Sciences. Different source accelerating voltages (50 to 80 kV) and powers (4 to 9 W) were used depending on fragment size for XRM analyses. Projections of each fragment were obtained by rotating this fragment around its axes in an angular range from -180° to 180° with increments of 0.1°. The objective magnification was set to 4 times. The exposure time of each projection was 1 or 2 seconds depending on fragment size. Therefore, the total number of projections for each fragment was between 1001 and 2801. The spatial resolution of each sample scan was approximately 1.12 μm. The acquired voxel data were segmented and rendered using VG Studio MAX 3.5 software to obtain the relative volumes of different minerals in three fragments (Tables S1 and S2). The 3D textures of CE6 fragments were presented using three-dimensional reconstruction animation (Supplementary Videos 1–3).

**Petrographic observation**

Seven polished thick sections were observed using a Zeiss Sigma 300 FE-SEM at State Key Laboratory of Critical Earth Material Cycling and Mineral Deposits, Nanjing University, and a Tescan MIRA3 FE-SEM at Nanjing Hongchuang Geological Exploration Technology Service Co., Ltd. SEM analyses using the Zeiss instrument were performed with an accelerating voltage of 20 kV and a working distance of 10.8 mm, while observations using the Tescan system were conducted with an accelerating voltage of 25 kV and a working distance of 11.9 mm. Energy dispersive X-ray spectroscopy (EDS) was used to identify mineral phases in each CE6 lithic fragment. Combined with backscattered electron (BSE) images (Figs. 1, S1, and S2), modal abundances of different mineral phases were obtained. A total of 37 highlands clasts and 10 basaltic clasts were identified based on their petrographic textures and mineral compositions (Figs. 1, S1, and S2, Tables S1 and S2).

**Major element analysis**

Major element compositions of different minerals in 37 highlands clasts and 10 basalt clasts were analyzed using a JEOL JXA-8230 and a JEOL JXA-8530 electron probe microanalyzer at State Key Laboratory of Critical Earth Material Cycling and Mineral Deposits, Nanjing University, and a JEOL JXA-iSP100 at Nanjing Hongchuang Geological Exploration Technology Service Co., Ltd. Each analysis was performed with an accelerating voltage of 15 kV and a beam current of 20 nA. The analytical spot size varied from 1 to 10 µm depending on the grain size. The standards used in this study were natural and synthetic crystals. All collected EPMA data were subjected to ZAF correction. The analytical uncertainties were better than 1–3%. Bulk compositions of CE6 basalts were calculated using mineral EPMA data (Supplementary Table 1) and mineral modal abundances of these clasts (Tables S2 and S3).

**Trace element analysis**

Trace element compositions of pyroxene and plagioclase (Supplementary Table 2) were analyzed using a Thermo Scientific iCAP Q quadrupole inductively coupled plasma mass spectrometer coupled with an ASI RESOlution S-155 193nm ArF excimer laser (LA-ICP-MS) at State Key Laboratory of Critical Earth Material Cycling and Mineral Deposits, Nanjing University. Trace element analyses were carried out using a laser beam diameter of 29 μm, a beam energy density of ~4.0 J/cm^2^, and a repetition rate of 5 Hz. Each analysis included 20 s background collection, 40 s sample ablation, and 25 s washing time. Reference glass NIST 610 was analyzed as an external standard, while reference glasses NIST 612, BCR-2G, and GSE-1G were used as monitoring standards. ^29^Si was used as an internal standard to correct for instrument drift. Data processing was conducted using ICPMSData version 12.2 [1]. The analytical precision was better than 10%.

**U-Pb isotope analysis**

Lead isotopic compositions of CE6 basalt samples were analyzed using a CAMECA 1300-HR^3^ ion microprobe at State Key Laboratory of Critical Earth Material Cycling and Mineral Deposits, Nanjing University. An ^16^O^-^ primary beam with different currents was focused into spots with sizes of < 3 μm to ~10 μm using Gaussian illumination mode. Prior to each analysis, pre-sputtering (80–150 s) was performed on the target area to remove the conductive coating and potential contaminants. A multi-collector mode with four electron multipliers (EM) was used to collect secondary ions ^204^Pb^+^ (L2), ^206^Pb^+^ (L1), ^207^Pb^+^ (C), and ^208^Pb^+^ (H2) in the first step with 30 s counting time for all except ^208^Pb^+^ (10 s), and ^238^U^16^O^+^ (H2) was collected in the second step with 4 s counting time in each cycle. Ion images with ^96^Zr_2_^16^O_2_^+^ or Ca_2_PO_3_^+^ on approximately 30×30 μm² area were used to precisely locate the target minerals when analyzing Zr-bearing minerals or phosphates, respectively. The signal of ^208^Pb^+^ was used for peak centering. High-purity oxygen gas was leaked to sample surface to enhance Pb^+^ yield during all sessions [2]. The mass resolution power (MPR) values were around 7000.

Different phases in CE6 clasts were analyzed in sessions B1 and B2. In session B1, Zr-bearing minerals, phosphates, K-feldspar, ilmenite, and matrix were analyzed using a small beam current (2 nA, 0.3 nA, or 0.15 nA) and a spot size of ≤3 μm, and each analysis consisted of 20 cycles. In session B2, matrix and silicate minerals were analyzed using a 10 nA current and a spot size of ~10 μm, and each analysis consisted of 20 cycles. Glass standards BCR-2G, NIST 610, and BHVO-2G were analyzed to monitor the instrument stability during sessions A and B. The IMF correction was independently performed for each analytical session. Analyses in both sessions were corrected using standard BCR-2G [3]. Data (Supplementary Table 3) were processed using in-house SIMS data reduction spreadsheets and Excel add-in Isoplot (Version 4.15) [4].

The mantle source μ value (^238^U/^204^Pb; μ_2_) was calculated using a two-stage Pb evolution mode [5, 6]. This model assumes that distinct silicate reservoirs formed during LMO crystallization, which were mantle sources for various basaltic rocks. Given that the leftmost Pb-Pb isochrons of basalt clasts F29, F42, and F34 exhibit consistent slopes and y‑intercepts (radiogenic ^207^Pb/^206^Pb ratios) within uncertainty, the integrated isochron of these clasts is considered to reliably represent the μ‑value of the CE6 basalt mantle source. This integrated isochron (including two points from clast F28), constructed using Zr-bearing, phosphate, and silicate minerals, has a slope of 150.0±8.7 and a y-intercept of 0.1983±0.0006 (Fig. 2a). We adopted a Moon formation time (t_0_) of 4500 Ma, with a differentiation time (t_1_) of 4376±18 Ma, and a common μ value (μ_1_ = 462±42) for a single primordial lunar reservoir [5]. Our result constrains the mantle source μ value to 330±39.

**In-situ Sr isotope analysis**

Strontium isotopic compositions of plagioclase in CE6 basalts were determined using a Neptune Plus multi-collector inductively coupled plasma mass spectrometer coupled with a Geolas Pro laser ablation system (LA-MC-ICP-MS) at State Key Laboratory of Critical Earth Material Cycling and Mineral Deposits, Nanjing University. Analyses were performed with a beam size of 60 µm, a laser frequency of 6 Hz, and an energy density of 10 J/cm^2^. Reference glass NIST 610 was used to tune the instrumental sensitivity before sample measurement. The signal was acquired with a collection time of 60 s after a 30 s blank collection. The potential isobaric interferences (Kr, Yb^2+^, Er^2+^, and Rb^+^) were evaluated and corrected using the established protocols [7]. The Sr isotopic data were corrected for mass fractionation using ^86^Sr/^88^Sr ratio of 0.1194 by exponential law [7]. Reference anorthite YG 4301 [8] was analyzed as an unknown to assess accuracy and reproducibility of Sr isotope determinations (Table S5). All isotopic data were processed by using an in-house Excel VBA (Visual Basic for Applications) reduction procedure.

**Trace-element modelling of petrogenesis**

Rayleigh fractionation was used to model trace element evolution in the residual melt during fractional crystallization following:

$\frac{C_{L}}{C_{0}}=F^{D-1}$	(1)

where *C_L_* is trace element concentration in the residual melt, *C*_0_ is trace element concentration in the parental melt, *F* denotes mass fraction of residual melt, and *D* is bulk partition coefficient, which can be calculated using modal abundance and partition coefficient of each crystallizing phase (Supplementary Table 4). The parental melt REE compositions of CE6 basalts were calculated (Table S4) using the most Mg-rich pyroxenes in the basaltic clasts (i.e., clast D19) with the respective partition coefficients (Supplementary Table 4).

Batch melting model was used to calculate REE concentrations in the partial melts of mantle source following:

$\frac{C_{L}}{C_{M}}=\frac{1}{D+F(1-D)}$	(2)

where *C_L_* is trace element concentration in the melt, *C_M_* is trace elemental concentration in the mantle source, *D* denotes bulk partition coefficient between mantle mineral and melt, and *F* denotes mass fraction of the partial melt. The REE compositions of the partial melts for CE6 basalts are shown in Table S4.

**Fig. S1 Backscattered electron images of CE6 highlands lithologies. a**, Gabbroic anorthosite (clast G03). **b**, Gabbroic anorthosite (clast H23). **c**, Anorthosite (clast H24). **d**, Anorthosite (clast H45). **e**, Anorthositic norite of Mg-suite (clast G04) **f**, Anorthositic norite of Mg-suite (clast H27). **g**, Anorthositic gabbro of Mg-suite (clast B09). **h**, Anorthositic gabbro of Mg-suite (clast H13). **i**, Anorthositic gabbro of Mg-suite (clast H28). Px, pyroxene; Pl, plagioclase; Ol, Olivine.

**Fig. S2 Backscattered electron images of CE6 low-Ti basalts.** **a**, clast F34. **b**, clast F38. **c**, clast F28. **d**, clast F06. **e**, clast F40. **f**, clast C01. Px, pyroxene; Pl, plagioclase; Ilm, ilmenite.

**Fig. S3 Mineral modal abundances and chemical compositions of CE6 highlands lithologies.** **a**, Mineral modes of CE6 highlands lithologies. Each highlands clast (Table S1) was classified using modal abundances of olivine, pyroxene, and plagioclase [9]. **b**, Major element compositions in pyroxene of CE6 highlands lithologies. **c**, Major element compositions in plagioclase of CE6 highlands lithologies. **d**, Correlation between Mg# of mafic minerals and An of plagioclase in highlands clasts (Table S1). Literature data of Apollo samples [10-14] are shown for comparison. A, anorthosite; NA, noritic/gabbroic anorthosite; TA, troctolitic anorthosite; AN, anorthositic norite/gabbro; AT, anorthositic troctolite; N, norite/gabbro; ON, olivine norite/gabbro; T, troctolite; P, pyroxenite; Pe, peridotite; D, dunite.

**Fig. S4 Mineral modal abundances and chemical compositions of CE6 low-Ti basalts.** **a**, Mineral modes of CE6 basalts (Table S2). **b**, Major element compositions in pyroxene of CE6 basalts. **c**, Major element compositions in plagioclase of CE6 basalts. **d**, Correlation between Ti# and Fe# in pyroxene of CE6 basalts. Literature data [11, 15] are shown for comparison.

**Fig. S5 Trace element compositions of CE6 low-Ti basalts.** **a**, Plagioclase REE distribution patterns in CE6 basalts. **b**, Pyroxene REE distribution patterns in CE6 basalts. Shaded areas represent REE distribution patterns in CE5 basalts [16]. **c**, Incompatible trace element ratios in mare basalts. CE6 data represent the trace element ratios of melt in equilibrium with the most magnesian pyroxene in each clast. Literature CE5 and Apollo data are from [15, 16] and Clive Neal's Mare Basalt Database (https://www3.nd.edu/~cneal/Lunar-L/). **d**, Petrogenetic modelling of CE6 basalts. Black solid line with diamonds represents parental melt of CE6 basalts, which could have formed through 2–3% partial melting of 86 PCS (percent crystallized solid) + 1% TIRL (trapped instantaneous residual liquid) (Table S4) and subsequent fractional crystallization. Literature data of Apollo high-Ti source and mantle source of 86PCS + 2% TIRL [17, 18] are shown for comparison.

**Fig. S6 Pb-Pb isochron ages of CE6 low-Ti basalts. a**, Pb-Pb isotope compositions of CE6 basalts. Solid symbols represent analyses used to define the leftmost Pb-Pb isochron. Open symbols represent the analyses affected by terrestrial contamination. Triangle area represents mixing between initial Pb, radiogenic Pb, and modern terrestrial Pb isotope compositions [19]. **b**, Pb-Pb isochron of clast F34. **c**, Pb-Pb isochron of clast F29. **d**, Pb-Pb isochron of clast F42. Error bars represent 2σ.

**Table S1 Mineral modes, plagioclase An, and mafic mineral Mg# in CE6 highlands lithologies.**

| **Clast** | **Lithology** | **Plagioclase** | | **Pyroxene** | **Low-Ca**  **Pyroxene** | **Olivine** | |
| --- | --- | --- | --- | --- | --- | --- | --- |
|  |  | **vol%** | **An** | **vol%** | **Mg#** | **vol%** | **Mg#** |
| **A06-X2** | Gabbroic anorthosite | 84.1 | 94.52 | 15.9 | 63.23 |  |  |
| **B09^a^** | AG of Mg-suite | 70.8 | 94.53 | 29.2 | 62.03 | trace | 60.24 |
| **B09** | AG of Mg-suite | 69.4 | 94.53 | 30.5 | 62.03 | trace | 60.24 |
| **C06-X3** | Anorthosite | 90.9 | 95.37 | 7.2 | 61.37 | 1.9 | 56.98 |
| **D10-X2** | AN of Mg-suite | 75.0 | 96.25 | 12.2 | 74.31 | 12.8 | 70.16 |
| **D17** | AN of Mg-suite | 73.0 | 95.74 | 14.2 | 88.31 | 12.8 |  |
| **F23-X1** | AG of Mg-suite | 75.4 | 95.25 | 24.6 | 61.94 |  |  |
| **F30** | Gabbroic anorthosite | 88.5 | 96.40 | 11.5 | 66.19 |  | 56.41 |
| **F35** | AG of Mg-suite | 64.7 | 92.81 | 35.3 | 64.88 |  |  |
| **F44-X5** | AN of Mg-suite | 69.1 | 97.20 | 30.9 | 78.11 |  |  |
| **G03** | Gabbroic anorthosite | 83.6 | 96.71 | 14.8 | 66.84 | 1.6 | 57.17 |
| **G04** | AN of Mg-suite | 72.2 | 95.57 | 27.8 | 69.99 |  |  |
| **G05** | AN of Mg-suite | 63.8 | 96.29 | 36.2 | 78.46 |  |  |
| **G14** | Anorthosite | 90.5 | 96.43 | 9.5 | 68.73 |  |  |
| **G25** | AG of Mg-suite | 79.2 | 95.97 | 13.7 | 78.62 | 7.1 | 77.16 |
| **G34** | AG of Mg-suite | 77.6 | 96.74 | 13.9 | 78.80 | 8.5 | 77.40 |
| **G38** | AG of Mg-suite | 74.5 | 95.78 | 25.5 | 61.52 |  |  |
| **G43** | AG of Mg-suite | 65.9 | 94.98 | 31.2 | 70.33 | 3.0 | 58.05 |
| **H02** | AG of Mg-suite | 67.9 | 94.81 | 32.1 | 61.06 |  |  |
| **H06** | AG of Mg-suite | 78.9 | 96.75 | 19.9 | 84.81 | 1.3 | 69.37 |
| **H10** | Anorthosite | 91.5 | 96.97 | 5.8 | 66.37 | 2.7 | 57.18 |
| **H11** | Anorthosite | 90.3 | 97.88 | 9.6 | 67.54 | trace |  |
| **H12** | AN of Mg-suite | 69.8 | 98.47 | 25.4 | 77.43 | 4.8 |  |
| **H13** | AG of Mg-suite | 79.5 | 96.21 | 13.2 | 76.45 | 7.4 | 69.20 |
| **H14** | Anorthosite | 90.1 | 95.71 | 9.7 | 65.00 | 0.2 | 56.78 |
| **H17** | AG of Mg-suite | 76.0 | 96.25 | 20.5 | 78.30 | 3.5 | 78.04 |
| **H18** | Gabbroic anorthosite | 85.2 | 96.67 | 14.2 | 66.51 | 0.6 | 58.40 |
| **H19** | Gabbroic anorthosite | 87.8 | 96.13 | 10.7 |  | 1.6 | 56.75 |
| **H23** | Gabbroic anorthosite | 87.2 | 96.32 | 12.6 | 66.37 | 0.3 | 56.95 |
| **H24** | Anorthosite | 90.7 | 97.01 | 9.1 | 66.70 | 0.2 | 57.50 |
| **H27** | AN of Mg-suite | 67.1 | 96.75 | 22.9 | 81.03 | 10.0 | 62.40 |
| **H28** | AG of Mg-suite | 77.8 | 96.14 | 12.4 | 78.82 | 9.8 | 76.92 |
| **H29** | Anorthosite | 93.4 | 97.05 | 6.6 |  | 0.1 | 55.20 |
| **H33** | Noritic anorthosite | 84.2 | 96.25 | 15.7 | 63.35 |  |  |
| **H40** | AG of Mg-suite | 77.8 | 94.93 | 16.8 | 79.17 | 5.5 | 77.36 |
| **H45** | Anorthosite | 93.1 | 97.93 | 5.3 | 64.82 | 1.6 | 54.95 |
| **H46** | Troctolitic anorthosite | 89.7 | 97.34 | 0.8 | 73.22 | 9.5 | 71.43 |
| **H51** | AN of Mg-suite | 79.0 | 95.67 | 20.8 | 77.85 | 0.2 |  |

^a^Modal abundances of mineral phases were determined using XRM voxel data. Both XRM voxel data and BSE pixel data were used to determine mineral modes in clast B09. Mineral shapes, sizes, and distributions shown in 3D reconstruction of clast B09 (Supplementary Video 1) are more precise than those observed in BSE image (Fig. S1g) and thus modal abundances calculated using XRM voxel data were used in this study. AN, anorthositic norite; AG, anorthositic gabbro.

**Table S2 Mineral modes of CE6 low-Ti basalts.**

| **Clast** | **Plagioclase** | **Pyroxene** | **Ilmenite** | **Olivine** | **Quartz** | |
| --- | --- | --- | --- | --- | --- | --- |
|  | **vol%** | **vol%** | **vol%** | **vol%** | **vol%** | |
| B03^a^ | 27.9 | 65.9 | 6.2 |  |  | |
| B03 | 31.0 | 61.6 | 7.4 |  |  | |
| C01^a^ | 32.9 | 61.5 | 5.6 |  |  |  |
| C01 | 44.4 | 50.4 | 5.2 |  |  |  |
| F06 | 44.7 | 46.1 | 5.3 | 3.7 | 0.2 | |
| F28 | 37.8 | 58.1 | 4.1 |  |  |  |
| F38 | 37.9 | 58.0 | 4.1 |  |  | |
| F40 | 43.6 | 46.9 | 5.2 | 4.4 |  | |
| F42 | 33.0 | 59.9 | 7.2 |  |  |  |
| G44 | 37.3 | 54.7 | 7.3 |  | 0.6 | |

^a^Modal abundances of mineral phases were calculated using XRM voxel data (Supplementary Videos 2-3), which were used for these clasts in this study.

**Table S3 Bulk clast compositions of CE6 low-Ti basalts.**

| **Clast** | **SiO_2_** | **TiO_2_** | **Al_2_O_3_** | **Cr_2_O_3_** | **FeO** | **MnO** | **MgO** | **CaO** | **Na_2_O** | **K_2_O** | **Total** |
| --- | --- | --- | --- | --- | --- | --- | --- | --- | --- | --- | --- |
|  | **wt%** | **wt%** | **wt%** | **wt%** | **wt%** | **wt%** | **wt%** | **wt%** | **wt%** | **wt%** | **wt%** |
| B03^a^ | 44.11 | 6.00 | 9.68 | 0.34 | 19.77 | 0.25 | 7.00 | 12.45 | 0.38 |  | 99.98 |
| C01^a^ | 44.65 | 5.20 | 10.42 | 0.25 | 21.21 | 0.26 | 6.51 | 11.04 | 0.43 | 0.02 | 99.99 |
| F06 | 43.51 | 4.85 | 13.64 | 0.20 | 18.70 | 0.23 | 6.25 | 12.10 | 0.49 | 0.03 | 100.00 |
| F28 | 45.06 | 4.07 | 11.98 | 0.26 | 19.41 | 0.26 | 6.31 | 12.12 | 0.47 | 0.05 | 99.99 |
| F38 | 45.35 | 3.97 | 11.39 | 0.15 | 21.14 | 0.28 | 6.21 | 10.96 | 0.52 | 0.03 | 100.00 |
| F40 | 43.51 | 5.11 | 12.99 | 0.20 | 20.51 | 0.26 | 5.31 | 11.46 | 0.61 | 0.04 | 100.00 |
| F42 | 43.12 | 6.74 | 10.48 | 0.26 | 20.50 | 0.26 | 6.61 | 11.52 | 0.47 | 0.03 | 99.99 |
| G44 | 43.50 | 6.24 | 12.13 | 0.40 | 17.18 | 0.22 | 6.08 | 13.77 | 0.47 | 0.02 | 100.01 |

^a^Bulk clast compositions were calculated based on mineral modes determined using XRM voxel data (Table S2).

**Table S4 REE compositions of parental melt and mantle source for CE6 low-Ti basalts.**

|  | | Parental melt | Source^a^ | 2% partial melting^b^ | 3% partial melting^b^ |
| --- | --- | --- | --- | --- | --- |
| La | ppm | 3.74 | 0.08 | 3.19 | 2.27 |
| Ce | ppm | 12.25 | 0.21 | 7.75 | 5.68 |
| Pr | ppm |  |  |  |  |
| Nd | ppm | 11.55 | 0.18 | 5.17 | 4.05 |
| Sm | ppm | 3.92 | 0.07 | 1.53 | 1.27 |
| Eu | ppm | 0.39 | 0.02 | 0.25 | 0.22 |
| Gd | ppm | 4.23 | 0.12 | 2.07 | 1.78 |
| Tb | ppm | 0.85 | 0.03 | 0.39 | 0.34 |
| Dy | ppm | 5.13 | 0.20 | 2.68 | 2.37 |
| Ho | ppm |  |  |  |  |
| Er | ppm | 2.69 | 0.17 | 1.95 | 1.77 |
| Tm | ppm |  |  |  |  |
| Yb | ppm | 2.43 | 0.23 | 2.19 | 2.02 |
| Lu | ppm | 0.40 | 0.04 | 0.34 | 0.32 |

^a^Mantle source denotes 86PCS+1.0% TIRL [18].

^b^Compositions of mantle partial melts were calculated using Equation 2 with mineral modal abundances of 40% olivine, 23% orthopyroxene, 27% pigeonite, 8% augite, and 3% plagioclase.

**Table S5 Sr isotope compositions of plagioclase in CE6 low-Ti basalts.**

| **Sample** | **^87^Sr/^86^Sr** | **2SE** | **Initial ^87^Sr/^86^Sr** | **2SE** |
| --- | --- | --- | --- | --- |
| **B03** | 0.70025 | 0.00013 | 0.69951 | 0.00013 |
| **F06-1** | 0.69943 | 0.00012 | 0.69919 | 0.00012 |
| **F06-2** | 0.69961 | 0.00011 | 0.69926 | 0.00011 |
| **F28** | 0.6996 | 0.00013 | 0.69925 | 0.00013 |
| **F38-1** | 0.69959 | 0.00010 | 0.69925 | 0.00010 |
| **F38-2** | 0.69945 | 0.00011 | 0.69919 | 0.00011 |
| **F34-1** | 0.69944 | 0.00009 | 0.69919 | 0.00009 |
| **F34-2** | 0.69919 | 0.00013 | 0.69909 | 0.00013 |
| **F42** | 0.70005 | 0.00011 | 0.69943 | 0.00011 |
| **Standard** |  |  |  |  |
| **YG 4301 (N=12)** | 0.70343 | 0.00003 |  |  |

Reference value of standard YG 4301 is 0.70342±0.00003 [8].

**References**

1.	Liu Y, Hu Z, Gao S, et al. In situ analysis of major and trace elements of anhydrous minerals by LA-ICP-MS without applying an internal standard. *Chem Geol* 2008; **257**: 34–43.

2.	Li QL, Li XH, Liu Y, et al. Precise U-Pb and Pb-Pb dating of Phanerozoic baddeleyite by SIMS with oxygen flooding technique. *J Anal Atom Spectrom* 2010; **25**: 1107–1113.

3.	Elburg M, Vroon P, van der Wagt B, et al. Sr and Pb isotopic composition of five USGS glasses (BHVO-2G, BIR-1G, BCR-2G, TB-1G, NKT-1G). *Chem Geol* 2005; **223**: 196–207.

4.	Ludwig KR. *Isoplot 4.15: A geochronological toolkit for Microsoft Excel*. 2008.

5.	Snape JF, Nemchin AA, Bellucci JJ, et al. Lunar basalt chronology, mantle differentiation and implications for determining the age of the Moon. Earth Planet Sci Lett 2016; **451**: 149–158.

6.	Li QL, Zhou Q, Liu Y, et al. Two-billion-year-old volcanism on the Moon from Chang’e-5 basalts. *Nature* 2021; **600**: 54–58.

7.	Yang YH, Wu FY, Yang JH, et al. Sr and Nd isotopic compositions of apatite reference materials used in U-Th-Pb geochronology. *Chem Geol* 2014; **385**: 35–55.

8.	Zhang W, Hu Z, Liu Y, et al. Improved in situ Sr isotopic analysis by a 257 nm femtosecond laser in combination with the addition of nitrogen for geological minerals. *Chem Geol* 2018; **479**: 10–21.

9.	Lucey P, Korotev RL, Gillis JJ, et al. Understanding the lunar surface and space-Moon interactions. *Rev Mineral Geochem* 2006; **60**: 83–219.

10.	Papike JJ, Fowler GW, Shearer CK, et al. Ion microprobe investigation of plagioclase and orthopyroxene from lunar Mg-suite norites: Implications for calculating parental melt REE concentrations and for assessing postcrystallization REE redistribution. *Geochim Cosmochim Acta* 1996; **60**: 3967–3978.

11.	Papike JJ, Ryder G, Shearer CK. Lunar samples. *Rev Mineral Geochem* 1998; **36**: 5-1–5-234.

12.	Pernet-Fisher JF, Deloule E, Joy KH. Evidence of chemical heterogeneity within lunar anorthosite parental magmas. *Geochim Cosmochim Acta* 2019; **266**: 109–130.

13.	Shearer CK, Elardo SM, Petro NE, et al. Origin of the lunar highlands Mg-suite: An integrated petrology, geochemistry, chronology, and remote sensing perspective. *Am Mineral* 2015; **100**: 294–325.

14.	Torcivia MA, Neal CR. Unraveling the components within Apollo 16 ferroan anorthosite suite cataclastic anorthosite sample 60025: Implications for the lunar magma ocean model. *J Geophys Res Planets* 2022; **127**: e2020JE006799.

15.	Tian HC, Wang H, Chen Y, et al. Non-KREEP origin for Chang’e-5 basalts in the Procellarum KREEP Terrane. *Nature* 2021; **600**: 59–63.

16.	Jiang Y, Kang J, Liao S, et al. Fe and Mg isotope compositions indicate a hybrid mantle source for young Chang’E 5 mare basalts. Astrophys J Lett 2023; 945: L26.

17.	Hallis L, Anand M, Strekopytov S. Trace-element modelling of mare basalt parental melts: Implications for a heterogeneous lunar mantle. *Geochim Cosmochim Acta* 2014; **134**: 289–316.

18.	Snyder GA, Taylor LA, Neal CR. A chemical model for generating the sources of mare basalts: Combined equilibrium and fractional crystallization of the lunar magmasphere. *Geochim Cosmochim Acta* 1992; **56**: 3809–3823.

19.	Stacey JS, Kramers JD. Approximation of terrestrial lead isotope evolution by a two-stage model. *Earth Planet Sci Lett* 1975; **26**: 207–221.
